# Retraction Note: Bone mesenchymal stem cell-derivedexosomal microRNA-29b-3p preventshypoxic-ischemic injury in rat brain byactivating the PTEN-mediated Aktsignaling pathway

**DOI:** 10.1186/s12974-020-02034-6

**Published:** 2020-11-23

**Authors:** Kun Hou, Guichen Li, Jinchuan Zhao, Baofeng Xu, Yang Zhang, Jinlu Yu, Kan Xu

**Affiliations:** 1 Department of Neurosurgery, The First Hospital of Jilin University, No. 1Xinmin Avenue, Changchun, 130021 Jilin People’s Republic of China; 2 Department of Neurology, The First Hospital of Jilin University, Changchun, 130021 People’s Republic of China

**Retraction to: J Neuroinflammation 17, 46 (2020)**

**https://doi.org/10.1186/s12974-020-1725-8**

The authors have retracted this article [1] because they have been unable to replicate the results of their study. After publication they expanded the sample size and repeated the measurements, and found that miR-29b-3p was not always down-regulated in the rat MCAO model and OGD cell model. In addition, when the processing time was doubled, miR-29b-3p showed an upward trend. The conclusions presented are therefore not reliable. All authors agree with this retraction.
